# Two-Stage Meta-Learning with Matched Feature Regularization for Cross-Subject sEMG Gesture Recognition Under Posture Variation

**DOI:** 10.3390/s26175476

**Published:** 2026-08-29

**Authors:** Qi Li, Ying He, Anyuan Zhang

**Affiliations:** 1 Department of Computer Science and Technology, Changchun University of Science and Technology, No. 7186 Weixing Road, Changchun 130022, China; 2 Zhongshan Institute, Changchun University of Science and Technology, Zhongshan 528437, China

**Keywords:** surface electromyography, gesture recognition, cross-subject adaptation, meta-learning, data augmentation, feature regularization

## Abstract

Surface electromyography (sEMG)-based gesture recognition has attracted considerable attention in intelligent prosthesis control, human–computer interaction, and rehabilitation assistance. However, practical deployment remains challenging because new users can usually provide only a few labeled samples for calibration. Under cross-subject and posture-varying conditions, this setting further aggravates distribution shifts and can destabilize target-domain adaptation. To address these issues, this paper proposes a cross-subject sEMG gesture-recognition framework integrating training-time augmentation, two-stage meta-transfer learning, and matched feature regularization. Model-agnostic meta-learning (MAML) is first used on source-subject data to learn an initialization for rapid transfer, after which target-subject fine-tuning is performed with an auxiliary class-consistent feature constraint. Experiments were conducted on a self-collected 11-subject multi-posture dataset using a leave-one-subject-out (LOSO) protocol under FT-1_full, which uses one complete calibration repetition, and FT-2_k10, which uses 10 windows per gesture per posture. The proposed MAML + FT + MATCHED method achieved higher average Accuracy and Macro-F1 than Source-pretrain + FT. Under FT-2_k10, the average improvements were 9.20 and 9.70 percentage points, respectively. Nevertheless, the primary subject-level paired Wilcoxon comparisons did not reach statistical significance (Accuracy: *p* = 0.2402; Macro-F1: *p* = 0.2402; Holm-adjusted *p* = 1.0000 for both). The results therefore indicate favorable average trends and positive effect sizes, while pairwise statistical superiority was not established in the present 11-subject cohort.

## 1. Introduction

Surface electromyography (sEMG) is a non-invasive and wearable-friendly signal with broad application potential in intelligent prosthesis control, human–computer interaction, and rehabilitation assistance [1,2]. Although deep learning has greatly advanced sEMG-based gesture recognition under controlled conditions, practical deployment still faces major challenges in cross-user generalization, low-cost calibration, and robustness [2,3,4,5,6,7]. In cross-subject settings, differences in muscle anatomy, skin impedance, electrode placement, and exertion habits cause substantial distribution shifts, making it difficult for models trained on source subjects to generalize directly to unseen users [4,8]. Posture variation further exacerbates this problem by altering muscle recruitment patterns and signal statistics [9,10]. Moreover, new users usually provide only a few labeled samples for calibration, which increases the risk of overfitting and feature drift during fine-tuning. Therefore, achieving rapid few-shot cross-subject adaptation while maintaining robustness to posture variation remains a central challenge in sEMG gesture recognition.

To address these problems, previous studies have explored transfer learning, domain adaptation, and meta-learning. Transfer learning based on pretraining and fine-tuning can leverage source-subject data to learn generalizable representations and then adapt the model with a small amount of target-domain data [4,8]. Domain alignment, self-supervised pretraining, and adversarial adaptation have also been introduced to reduce distribution mismatch across subjects, sessions, and days [5,11,12]. Meanwhile, meta-learning explicitly trains a model to “learn how to adapt” through task-level optimization, and is therefore particularly suitable for cross-subject recognition when only limited labeled samples are available in the target domain [13,14]. In the EMG field, meta-learning and few-shot learning have already been applied to stroke rehabilitation intention inference, prosthetic recalibration, and gesture recognition, showing great potential in reducing user retraining cost [13,14,15,16]. However, most existing studies focus on single-posture, single-session, or standard public-dataset settings, while the problem of few-shot rapid adaptation under multi-posture cross-subject conditions remains insufficiently explored. Moreover, relying solely on meta-initialization and conventional fine-tuning is often insufficient to stabilize target-domain representations when the available target samples are extremely limited.

Besides transfer and adaptation mechanisms, data augmentation is another commonly used approach to improve the robustness of sEMG recognition systems. Noise perturbation, amplitude scaling, temporal shifting, and local masking can enlarge the training distribution and make the model more tolerant to acquisition variability [9,17]. Nevertheless, data augmentation alone cannot fully address rapid cross-subject adaptation. If auxiliary or matched samples are directly mixed into target-domain classification training at a large scale, they may disturb the target-domain distribution and weaken the specificity of few-shot fine-tuning [10]. Recent studies on diffusion-based augmentation, optimal sample matching, and prototype adaptation suggest that preserving target-domain representation structure while introducing auxiliary transfer information is important for robust adaptation [9,10,16,18].

Despite these advances, existing cross-subject sEMG adaptation methods still exhibit two major limitations in low-calibration scenarios. On the one hand, transfer learning and meta-learning can provide a favorable initialization for rapid adaptation, but they do not explicitly constrain how target-domain representations evolve during few-shot fine-tuning, which may lead to unstable updates under severe inter-subject and posture-induced distribution shifts [4,8,13,14,15,16]. On the other hand, although data augmentation improves robustness by enlarging the input distribution, it does not directly preserve the structural consistency of target-domain features [9,17]. These observations suggest that rapid adaptation and representation stabilization should be considered jointly rather than independently. Inspired by prior studies on prototype adaptation, optimal sample matching, and feature-level alignment [10,16,18], we hypothesize that combining a meta-learned initialization with class-consistent feature regularization based on matched source-domain reference samples can stabilize target-domain adaptation under few-shot conditions without substantially disturbing the dominant target-domain classification objective.

Based on this motivation, this paper proposes a robust cross-subject sEMG gesture recognition framework that integrates data augmentation, two-stage meta-transfer learning, and matched feature regularization. First, data augmentation is introduced during training to enlarge the coverage of the sample distribution and improve robustness to signal perturbations and posture variation. Second, stage-one meta-training based on model-agnostic meta-learning (MAML) is conducted on source-subject data to learn an initialization that can be rapidly transferred to unseen subjects. Finally, stage-two fine-tuning is performed on a few labeled samples from the target subject, where a matched feature regularization mechanism is introduced. Instead of using auxiliary samples as direct classification supervision, only class-consistent matched samples are used to impose an auxiliary feature-space constraint on target-domain representations, thereby alleviating feature drift during few-shot fine-tuning. Unlike methods that directly treat auxiliary samples as the main classification data, the matched mechanism in this work is designed primarily to stabilize feature representations without substantially altering the dominant distribution of target-domain learning.

The results show that the proposed method achieves higher average Accuracy and Macro-F1 under the present protocol, with lower across-subject variability in several comparisons. These observations are interpreted as favorable performance trends rather than conclusive pairwise superiority because the primary subject-level Wilcoxon comparisons did not reach statistical significance.

It should be noted that previous cross-subject and few-shot sEMG studies have adopted different datasets, gesture sets, subject partitions, and adaptation protocols, making direct numerical comparison across studies difficult. Therefore, rather than directly ranking performance values obtained under heterogeneous experimental settings, the present study evaluates all compared methods under the same LOSO protocol and reports both Accuracy and Macro-F1. In particular, Macro-F1 is included as a class-balanced metric complementary to overall Accuracy, providing a more comprehensive evaluation of multi-class gesture recognition performance.

The main contributions of this work are summarized as follows:

(1) We investigate the more realistic and challenging problem of multi-posture cross-subject sEMG gesture recognition, which is more application-oriented than conventional single-posture settings.
(2) We construct a two-stage rapid adaptation framework consisting of meta-learning pretraining and target-subject fine-tuning, enabling personalized adaptation under limited calibration samples.
(3) We propose a matched feature regularization mechanism that uses class-consistent matched samples as an auxiliary feature-space constraint during target-subject fine-tuning. Its empirical contribution is evaluated separately from the MAML initialization.
(4) We conduct systematic experiments on a self-collected multi-posture sEMG dataset and report subject-level paired inference, effect sizes, confidence intervals, and individual-subject observations to characterize both the average performance trends and their uncertainty.

The remainder of this paper is organized as follows. Section 2 reviews related work. Section 3 presents the proposed method, including signal preprocessing, data augmentation, the two-stage training framework, and matched feature regularization. Section 4 describes the experimental settings. Section 5 reports the experimental results and discussion. Finally, Section 6 concludes the paper and outlines future directions.

## 2. Related Work

### 2.1. Deep Learning for sEMG Gesture Recognition

With the rapid development of deep learning for temporal signal modeling, sEMG-based gesture recognition has gradually shifted from traditional handcrafted feature design to end-to-end representation learning. Early studies demonstrated that convolutional neural networks can effectively model local temporal patterns and inter-channel coupling in multi-channel EMG signals. Atzori et al. validated the effectiveness of convolutional networks on large-scale public EMG datasets and systematically analyzed the influence of preprocessing strategies, network size, and data augmentation on recognition performance [19,20]. Subsequently, researchers further investigated multi-channel temporal correlation, local muscle response patterns, and cross-scale dynamic features, leading to a variety of deep architectures, including compact CNNs [21,22], multi-scale convolutional models, temporal convolutional networks, CNN-RNN hybrids, and attention-enhanced structures [23,24,25,26,27].

From the development trend of existing studies, deep learning methods have significantly improved automatic feature extraction capability in sEMG gesture recognition. One line of work emphasizes directly learning discriminative representations from raw signals or sliding-window segments, thereby reducing the dependence on handcrafted time-frequency features [23,24]. Another line of work combines multi-stream fusion, dual-branch designs, convolutional and Transformer-based architectures, or lightweight attention modules to jointly capture local temporal changes and global channel dependencies, thus balancing recognition accuracy and deployment efficiency. For example, DSRANet adopts depthwise separable residual convolution and multi-axis attention mechanisms to achieve high recognition accuracy with a relatively small number of parameters [3]. CNN-Transformer and Vision Transformer-based hybrid approaches further strengthen the modeling of long-range dependency and complex time-frequency structures [25,26,27,28,29]. However, most of these methods are evaluated under fixed-subject, fixed-condition, or standard public-dataset settings, and still lack unified and targeted solutions for cross-subject transfer, few-shot personalized adaptation, and posture-induced distribution shifts.

### 2.2. Transfer Learning and Meta-Learning for Cross-Subject Adaptation

To cope with significant inter-subject variability, transfer learning has become an important direction in EMG research. Existing studies have explored pretraining and fine-tuning, domain adaptation, multi-source transfer, and federated transfer learning [2,4,8]. Fratti et al. [23] proposed a multi-scale CNN with pretraining strategies to improve rapid adaptation to new users. Li et al. [4] introduced an end-to-end transfer learning framework that significantly improved gesture recognition under both cross-subject and cross-day settings. In addition, privacy-preserving federated transfer learning has also been applied to sEMG gesture recognition to improve cross-user generalization without sharing raw data [8].

Beyond conventional pretraining and fine-tuning, domain adaptation methods have also been widely used to reduce feature shifts across subjects and sessions [30,31]. Existing work includes pairwise domain alignment, self-supervised pretraining combined with adversarial alignment, iterative self-training, and dynamic domain generalization. Although these approaches are effective in reducing source-target discrepancy, they often rely on extra adaptation branches, discriminators, or relatively complex training pipelines. When only a few labeled samples are available in the target domain, they may still suffer from unstable adaptation or high training cost.

In contrast, meta-learning explicitly optimizes the model’s rapid adaptation capability through task-level training, making it particularly suitable for cross-subject sEMG recognition when only a few labeled target samples are available [13,16,32]. La Rotta et al. [13] and Proroković et al. [14] demonstrated the effectiveness of MAML-based fast adaptation in stroke rehabilitation intention inference and prosthetic recalibration, respectively. In gesture recognition, few-shot learning has been introduced into EMG-based gesture classification, showing promising generalization ability to new subjects, repetitions, and gestures [15]. Lee et al. [16] further combined prototype learning with efficient meta-learning updates and obtained stronger performance under cross-session and cross-user settings. However, most existing meta-learning approaches focus more on “how to adapt quickly” than on “how to stabilize feature representations and suppress representation drift during few-shot target fine-tuning,” which becomes even more critical under posture-induced distribution shifts.

### 2.3. Data Augmentation and Feature-Level Constraints

Besides transfer and adaptation, data augmentation is another important strategy for improving the robustness of sEMG models. Prior studies have shown that appropriate training-time augmentation can expand the coverage of the sample distribution and reduce overfitting to specific acquisition conditions [17,19]. Later studies further combined noise perturbation, amplitude scaling, temporal shifting, and local masking on raw EMG sequences or their time-frequency representations. Self-supervised learning has also been introduced into sEMG representation modeling. VICReg-based studies suggest that self-supervised pretraining can provide a robust initialization for continuous dynamic EMG pattern recognition [33]. In more complex distribution-shift scenarios, diffusion models have been applied to cross-day HD-sEMG recognition by generating auxiliary samples [9].

Compared with input-level augmentation, feature-level regularization and distribution alignment focus more on reducing representational discrepancy between source and target domains [34]. Existing studies have proposed deep domain adaptation, subspace alignment, optimal transport, adversarial feature disentanglement, source-free domain adaptation, and metric-learning-based robust representation modeling. Although these approaches are effective in improving inter-domain consistency, they usually involve additional adaptation modules, discriminators, or complex training procedures. Different from these heavy-weight alignment strategies, this work focuses on the stability of few-shot fine-tuning. Therefore, we adopt a training-time matched feature regularization strategy: matched samples are not incorporated into the main classification training at a large scale, but are only used to impose auxiliary constraints in the feature space, thereby reducing representation drift while maintaining the relative stability of the target-domain main classification distribution. This idea complements existing heavy domain-alignment methods and is particularly suitable for multi-posture few-shot target adaptation [35].

## 3. Method

### 3.1. Problem Definition

This work focused on sEMG gesture recognition under cross-subject and posture-varying conditions. Let the source-domain dataset be denoted as

(1) $$D_{S}={\{(x_{i},y_{i},p_{i})\}}_{i=1}^{N_{S}},$$

where $N_{S}$ denotes the total number of samples in the source-domain dataset, $x_{i}\in R^{C\times T}$ denotes the i-th multi-channel sEMG segment, *C* denotes the number of sEMG channels, and *T* denotes the temporal length in samples. In this study, *C* = 8, *T* = 150 samples, and *K* = 8. At the sampling rate of 500 Hz, *T* = 150 samples corresponds to a temporal duration of 300 ms. $y_{i}\in \{1,\ldots ,K\}$ denotes the gesture label, and $p_{i}\in P$ denotes the posture label.

(2) P={P1,P2,P3,P4}.

For the target subject, the few labeled samples are denoted by

(3) $$D_{t}^{\sup}={\{(x_{j}\;,y_{j}\;,p_{j})\}}_{j=1}^{N_{t}^{\sup}},$$

where the superscript “sup” denotes the support set used for target-subject adaptation, and $N_{t}^{\sup}$ denotes the number of labeled target-domain samples in this support set, while the remaining samples constitute the test set.

(4) $$D_{t}^{\mathrm{qry}}={\{(x_{m},y_{m},p_{m})\}}_{m=1}^{N_{t}^{\mathrm{qry}}}.$$

where the superscript “qry” denotes the query set, which contains the remaining target-subject samples used for final evaluation, and $N_{t}^{\mathrm{qry}}$ denotes the number of samples in this query set.

The goal of this paper was to learn a transferable initialization from multiple source subjects and rapidly adapt the model to a new target subject using only a small number of labeled samples, so as to achieve high recognition performance on $D_{t}^{\mathrm{qry}}$. To this end, we proposed a robust recognition framework integrating data augmentation, two-stage meta-transfer learning, and matched feature regularization.

### 3.2. Signal Preprocessing and Segmentation

The raw sEMG signals were first digitally filtered to suppress noise and then standardized to reduce overall amplitude-scale differences across recording conditions. A fourth-order Butterworth band-pass filter with an effective passband of 20–237.5 Hz and a 50 Hz notch filter (Q = 30) were applied. Both filters were applied using forward–backward zero-phase filtering.

For normalization, the filtered 5-s activity segments were grouped separately for each subject-posture condition. For each subject-posture condition, a single scalar mean $\mu_{s,p}$ and standard deviation $\sigma_{s,p}$ were calculated from the concatenated signal samples, where s and p denote the subject and posture, respectively. The same statistics were then used to standardize all signals within that subject-posture condition as

(5) $$x^{\prime }=\frac{x-\mu_{s,p}}{\sigma_{s,p}}$$

If $\sigma_{s,p}\;=\;0$, mean centering, $x^{\prime }\;=\;x-\mu_{s,p}$, was applied instead.

Then, a sliding-window strategy was employed to segment continuous action signals into fixed-length local samples for subsequent training and evaluation. Let the window length be *W*, and the step size be S. The raw sequence was segmented into a set of local samples

(6) $$\{x^{\left(1\right)},x^{\left(2\right)},{\ldots ,x}^{\left(M\right)}\},\;{\;x}^{\left(m\right)}\in R^{C\times W}.$$

In our experiments, the window length was set to *W* = 150 and the step size was set to *S* = 20. At a sampling rate of 500 Hz, this corresponds to a temporal window of approximately 300 ms and a step interval of approximately 40 ms. This setting preserved local dynamic patterns while providing a sufficient number of training samples.

Sliding-window segmentation served two purposes. First, it transformed long continuous signals into samples with a unified input size, which facilitated batch training. Second, it preserved short-term temporal dynamics of sEMG signals and thus improved the model’s ability to capture local time-varying characteristics. After preprocessing and segmentation, each sample was represented as a *C × W* tensor and was fed into the backbone network for feature extraction and classification.

### 3.3. Backbone Network

A one-dimensional convolutional neural network (1D-CNN) was used as the predefined backbone of the proposed framework. The network accepts an 8 × 150 sEMG window and contains three convolutional stages followed by an adaptive average-pooling layer and a fully connected classification head.

Each convolutional block follows the sequence Conv1d → GroupNorm → ReLU → MaxPool1d → Dropout, with 8 groups used in all GroupNorm layers. The three blocks use channel dimensions of 8→64, 64→128, and 128→256, with convolutional kernel sizes of 15, 11, and 7, respectively. Their MaxPool1d configurations are (kernel size 3, stride 2), (kernel size 3, stride 2), and (kernel size 2, stride 2), with dropout rates of 0.3, 0.3, and 0.4. The feature extractor is followed by AdaptiveAvgPool1d(1), Linear(256→128) → ReLU → Dropout(0.5), Linear(128→64) → ReLU → Dropout(0.3), and a final Linear(64→8) classification layer. The resulting model contains approximately 370 K trainable parameters. The output can be written as

(7) $$\hat{y}=f_{\theta }\left(x\right),$$

where $f_{\theta }\left(x\right)$ denotes the classifier parameterized by θ.

The predefined backbone learns representations directly from segmented multi-channel signals while retaining intermediate feature outputs. In particular, the conv1 representation is exposed for the matched feature regularization used during Stage-II adaptation.

### 3.4. Data Augmentation Strategy

To enlarge the local training distribution and improve robustness to acquisition variability, three time-domain augmentation operations were used in the reported experiments: amplitude scaling, additive Gaussian noise, and temporal shifting. Amplitude scaling was sampled from U(0.85, 1.15) with probability 0.6; Gaussian noise was independently applied with probability $p_{noise}\;=\;0.6$, with a standard deviation of 0.02 times the input-window standard deviation; and a circular temporal shift of up to ±10 samples was applied with probability 0.5. Temporal masking was not activated in the reported configuration.

Let an input sEMG window be denoted by $x\in R^{C\times T}$, where *C* = 8 is the number of channels and *T* = 150 is the number of samples in each window. The augmented window is denoted by $\tilde{x}$.

(1) Amplitude scaling. Amplitude scaling was defined as $\tilde{x}=ax$, where
  (8) a~U(0.85,1.15)

This transformation was applied with a probability of $p_{scale}=0.6$. It was used to model moderate amplitude variation caused by differences in muscle contraction intensity, electrode–skin contact, and subject-specific signal magnitude. The restricted scaling range was selected to avoid changing the basic temporal morphology or gesture label of the original signal.

(2) Additive Gaussian noise. Noise augmentation was defined as $\tilde{x}=x+\epsilon$, where
  (9) $$\epsilon_{c,t}\sim N(0,\sigma_{n}^{2}),$$
  and the noise level was determined adaptively from the current input window:
  (10) $$\sigma_{n}=0.02\frac{1}{C}\sum_{C=1}^{C}{Std}_{t}(x_{c,t}),$$

Gaussian noise was applied with a probability of $p_{noise}=0.6$. This operation represents low-amplitude electronic noise and minor acquisition fluctuations rather than severe movement artifacts. Evident movement artifacts in the original recordings were discarded and recollected during data acquisition rather than simulated through augmentation.

(3) Temporal shifting. Temporal shifting was implemented by shifting the input window along the temporal axis:
  (11) $${\tilde{x}}_{c,t}=x_{c,(t-\delta )\;\;}mod\;T,$$
  where
  (12) $$\delta \sim U_{int}(-10,10).$$

This operation was applied with a probability of $p_{shift}=0.5$. At a sampling rate of 500 Hz, the maximum displacement of 10 samples corresponded to approximately 20 ms. Temporal shifting was intended to model small differences in sliding-window alignment and local gesture timing rather than changes in muscle physiology. Circular boundary handling was used in the implementation as a computational approximation, and the displacement was restricted to 10 samples, corresponding to only 6.7% of the 150-sample window.

The three transformations were sampled independently, and more than one operation could therefore be applied to the same training window. During Stage I, augmentation was applied only to the support samples used for inner-loop adaptation, whereas the query samples used for meta-optimization remained unchanged. During Stage II, augmentation was applied only to target-domain training samples. Validation samples, test samples, and matched source-domain samples were not augmented.

These operations were not intended to generate new posture-specific physiological patterns. Instead, they introduced controlled perturbations corresponding to common amplitude, noise, and temporal-alignment variability while preserving the original gesture label.

### 3.5. Stage I: MAML-Based Meta-Training

To enable fast adaptation to unseen subjects, Stage I employed first-order model-agnostic meta-learning (FOMAML). The first-order formulation omitted second-order derivatives during the outer update while retaining task-specific inner-loop adaptation.

A set of K-way few-shot classification tasks was constructed from source-subject data. For each task $T_{i}$, a support set and a query set are sampled from source subjects under multiple posture conditions:

(13) $$T_{i}=\{D_{i}^{\sup},D_{i}^{\mathrm{qry}}\}.$$

Here, the support set was used for task-specific adaptation and the query set was used for meta-optimization. The reported main setting used 8-way tasks with 10 support windows and 40 query windows per gesture under each of the four postures, corresponding to 320 support and 1280 query windows per task. Balanced posture sampling was used to avoid task-construction bias toward a specific posture.

FOMAML was trained for 80 epochs with 100 tasks per epoch, an inner-loop learning rate of 0.005, an outer-loop learning rate of 0.001, three inner-loop updates, and a meta-batch size of 4.

An expanded meta-task sampling setting was used only for the auxiliary AUG/MATCHED sensitivity analysis. It used 40 support and 120 query windows per gesture per posture, corresponding to 1280 support and 3840 query windows per task. This setting changes the Stage-I task-sampling size and is not a separate target-subject adaptation protocol.

For each task $T_{i}$ the model first performed one or several gradient update steps on the support set to obtain task-specific parameters:

(14) $${\theta^{\prime }}_{i}=\theta -\alpha \nabla_{\theta }L_{\sup}^{\left(i\right)}(f_{\theta }),$$

where α is the inner-loop learning rate and $L_{\sup}^{\left(i\right)}$ is the classification loss on the support set.

Then, the updated parameters ${\theta^{\prime }}_{i}$ were evaluated on the query set, and the global meta-parameters were optimized by aggregating the query losses across tasks:

(15) $$\theta \leftarrow \theta -\beta \nabla_{\theta }\sum_{i}L_{\mathrm{qry}}^{\left(i\right)}(f_{{\theta^{\prime }}_{i}}),$$

where β is the outer-loop learning rate. After repeated meta-training, the learned parameter θ served as a transferable initialization that could be rapidly adapted to a new target subject with only a few labeled samples.

### 3.6. Stage II: Fine-Tuning with Matched Feature Regularization

In the second stage, the meta-learned initialization was fine-tuned using a small number of labeled samples from the target subject in order to achieve subject-specific adaptation. Since few-shot adaptation is prone to overfitting and posture variation may destabilize target-domain representations, we introduced matched feature regularization during fine-tuning to constrain feature drift.

Given the target-subject training set $D^{\sup}$, the basic classification loss in the fine-tuning stage is defined as

(16) $$L_{\mathrm{ce}}=-\sum_{(x,y)\in D_{t}^{\sup}}y\log f_{\theta }(x),$$

where $f_{\theta }(x)$ denotes the predicted class probability distribution. This loss ensures that the model remains driven primarily by the target subject’s original classification objective and learns a subject-specific decision boundary.

To reduce feature drift during few-shot adaptation, a matched source-reference set was constructed for each LOSO fold. The target reference consisted only of rep0 from the target subject, while candidate windows were drawn from the source-training subjects across postures P1–P4. Candidates were restricted to the same gesture class as the target reference. For each class, temporal similarity was measured using dynamic time warping (DTW), frequency-domain similarity was measured using the mean-squared error between FFT-magnitude representations, the two distances were min–max normalized and equally weighted, and the top 8% of candidates were retained. The reported configuration used 50 matched windows per class. Target-test samples and labels were never used for matching. The matched samples contributed only to the feature-space regularization term during Stage II.

(17) $$\mu_{c}^{t}=\frac{1}{\left|B_{c}^{t}\right|}\sum_{x\in B_{c}^{t}}\;\phi_{l}(x),$$

(18) $$\mu_{c}^{m}=\frac{1}{\left|B_{c}^{m}\right|}\sum_{x\in B_{c}^{m}}\;\phi_{l}(x).$$

where $B_{c}^{t}$ and $B_{c}^{m}$ denote the subsets of target-domain and matched samples of class c in the current mini-batch, respectively.

The matched feature regularization term is then defined as

(19) $$L_{\mathrm{match}}=\sum_{c\in C_{\mathrm{overlap}}}{\left\|\mu_{c}^{t}-\mu_{c}^{m}\right\|}_{2}^{2},$$

where $C_{\mathrm{overlap}}$ denotes the set of classes that appear in both the target-domain and matched samples in the current batch.

The overall loss in Stage II is therefore given by

(20) $$L_{\mathrm{total}}=L_{\mathrm{ce}}+\lambda L_{\mathrm{match}},$$

where λ is the regularization weight. The constraint was applied to the conv1 feature representation with an initial weight $\lambda_{0}$ = 0.02. The weight was held at its initial value during the first five fine-tuning epochs and was then linearly annealed toward zero for the remainder of Stage II, allowing the target classification objective to dominate later adaptation.

It should be emphasized that the proposed matched mechanism differs from directly mixing auxiliary samples into classification training. The matched source windows provide only an auxiliary feature-level constraint during fine-tuning and are not used as target-domain classification samples. The target-test partition was not used for matching, training, validation, or epoch selection.

### 3.7. Overall Optimization Procedure

The overall optimization procedure of the proposed method can be summarized as follows:

(1) Preprocess the raw sEMG signals and segment them into fixed-length sliding windows.
(2) Apply augmentation to training samples to enlarge the distribution and improve robustness.
(3) Construct meta-tasks from source-subject multi-posture data and perform Stage I MAML-based meta-training to learn a transferable initialization.
(4) Use the learned initialization as the starting point for Stage II fine-tuning on a few labeled target-subject samples.
(5) Introduce matched feature regularization during fine-tuning to suppress feature drift in few-shot adaptation.
(6) Evaluate the adapted model on the target-subject test set using Accuracy and Macro-F1.

Through this design, the proposed framework improved cross-subject sEMG gesture recognition from three complementary aspects: data-level robustness enhancement through augmentation, initialization-level fast adaptation through meta-learning, and target-domain representation stabilization through matched feature regularization.

**Figure 1:**
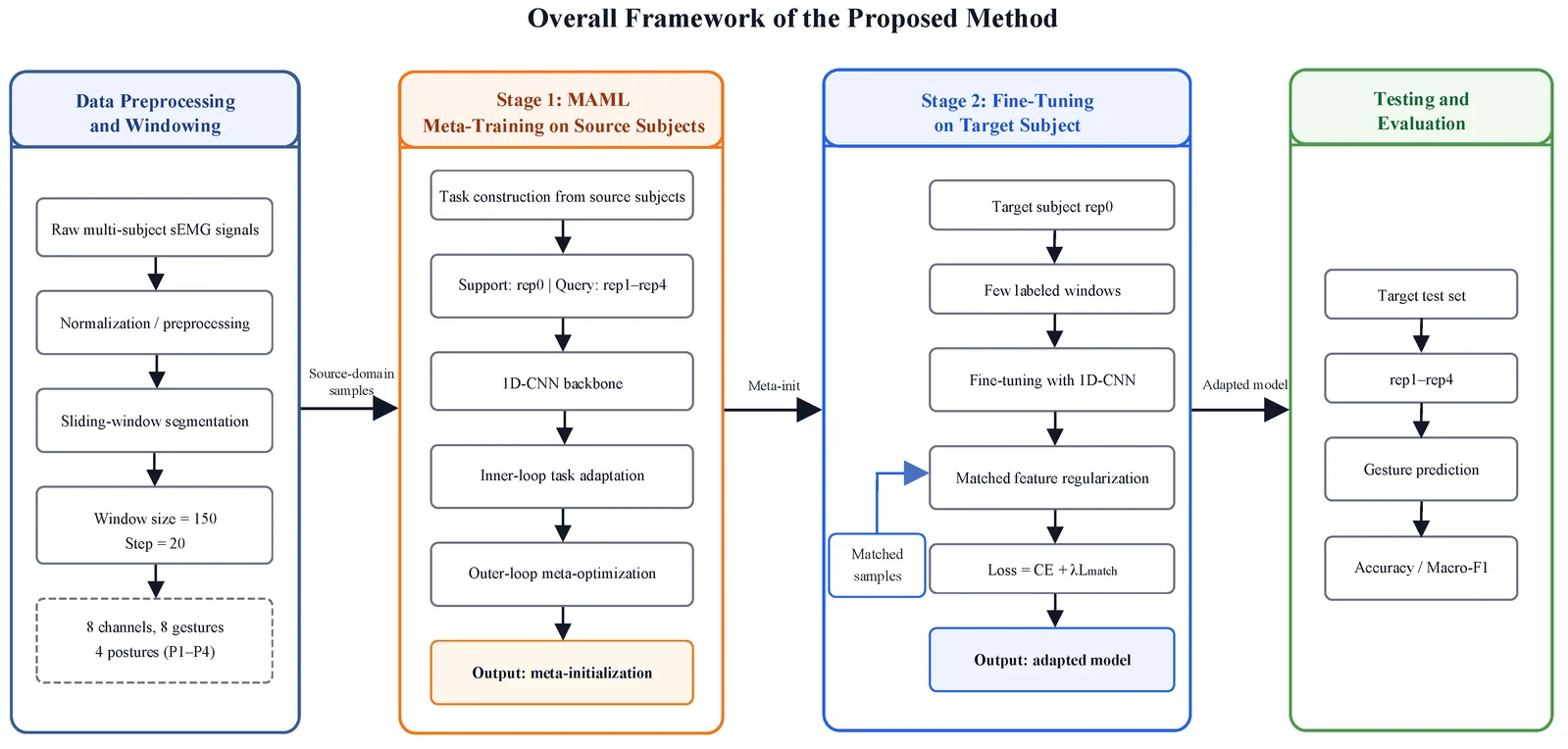
Overall framework of the proposed cross-subject sEMG gesture recognition method.

Figure 1 presents the overall framework of the proposed method. The complete workflow consists of four components: data preprocessing and sliding-window segmentation, Stage 1 MAML-based meta-training on source subjects, Stage 2 fine-tuning on the target subject with matched feature regularization, and final testing and evaluation on the remaining repetitions of the target subject. Specifically, the raw multi-subject sEMG signals were first normalized and segmented into fixed-length input samples through a sliding-window strategy. Meta-tasks were then constructed from the source-subject data, and MAML was employed to learn an initialization that was suitable for rapid transfer. Based on this initialization, the model was further fine-tuned using a small number of labeled windows from the target subject, while matched feature regularization was introduced to stabilize feature representations during few-shot adaptation. Finally, the adapted model was evaluated on the target-subject test set, with Accuracy and Macro-F1 adopted as the main evaluation metrics.

## 4. Experiment Setup

This section describes the dataset, evaluation protocol, implementation details, comparison methods, and evaluation metrics adopted in the experiments, thereby providing a unified experimental setup for the subsequent analysis of the results.

### 4.1. Dataset and Experimental Protocol

To validate the proposed method under cross-subject and posture-varying conditions, 11 healthy participants (nine males and two females, 22–27 years old) were recruited on campus. All participants were right-handed and reported no neuromuscular disorders or upper-limb injuries that could affect sEMG acquisition or gesture execution. Each participant received an explanation of the study purpose and procedures and provided written informed consent before data collection. The protocol was approved by the Ethics Committee of Changchun University of Science and Technology on 13 March 2026 (Approval No. 20260026). The sEMG signals were acquired using a commercially available eight-channel armband manufactured by Wuxi Sichiray Technology Co., Ltd. (Wuxi, China). The device used gold-plated dry electrodes measuring approximately 15 × 6 × 3 mm, arranged around the inner circumference at an approximately 20-mm center-to-center spacing, with differential acquisition and a shared reference electrode.

The eight sEMG channels were sampled at 500 Hz with a 12-bit resolution. According to the manufacturer documentation and subsequent technical confirmation, the acquisition circuit provided an amplification gain of 2000, a specified signal-to-noise ratio of 20 dB, and an input-referred voltage noise of 50 $nV/\sqrt{Hz}$. The acquired data were transmitted to the host computer through a Bluetooth 5.0 serial interface. The armband had an approximate diameter of 8 cm and an inner circumference of 23 cm.

Before data collection, the skin of the right forearm was cleaned with medical alcohol, and acquisition began only after the skin had completely dried. Visible sweat was removed when necessary, and the indoor temperature was kept stable throughout the experiment. The armband was worn on the right forearm, with its proximal edge positioned approximately 1.5 cm distal to the elbow crease. The armband remained attached during transitions among postures P1–P4. For participants with relatively thin forearms, the armband was additionally secured using its elastic structure or an auxiliary strap to reduce electrode displacement. Trials containing evident movement artifacts or abnormal signal fluctuations were discarded and recollected. Each gesture was repeated five times, with each repetition lasting approximately 7 s. Short resting intervals were provided between repetitions of the same gesture, while approximately 2-min rest intervals were provided between different gestures and between posture conditions to reduce muscle fatigue and changes in electrode–skin contact.

The acquisition device used a proprietary and non-open-source analog front end. Although the manufacturer was contacted for further technical details, the exact amplifier input impedance, common-mode rejection ratio, and pre-ADC analog filtering parameters were not disclosed. Therefore, these unavailable specifications were not inferred or replaced with parameters from other devices.

**Figure 2:**
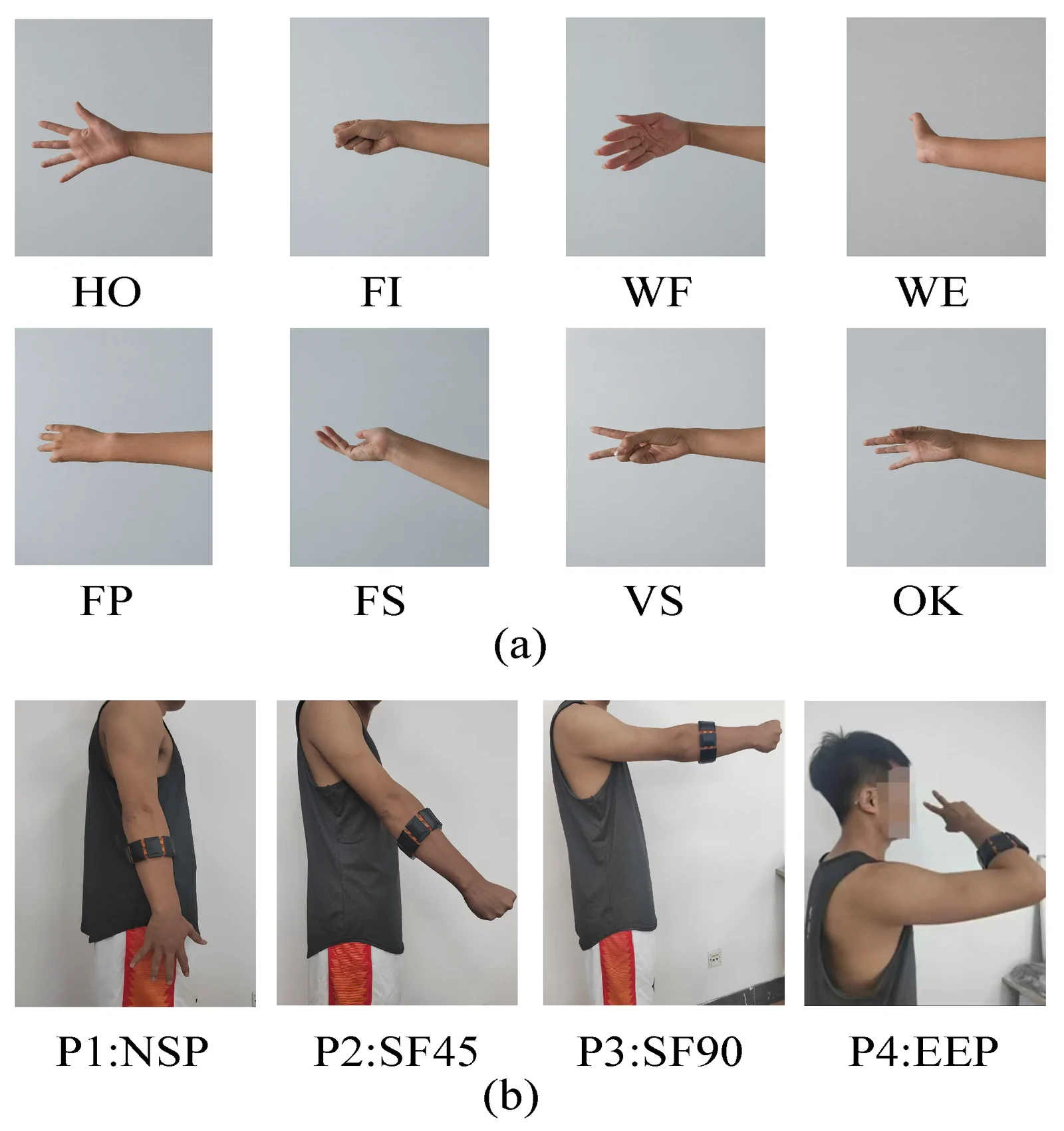
Illustration of the eight gesture classes and four posture conditions in the self-collected sEMG dataset. (**a**) Eight gesture classes: Hand Open (HO), Fist (FI), Wrist Flexion (WF), Wrist Extension (WE), Forearm Pronation (FP), Forearm Supination (FS), V-sign (VS), and OK Sign (OK). (**b**) Four posture conditions: neutral standing posture (NSP), 45° shoulder flexion (SF45), 90° shoulder flexion (SF90), and elbow-flexed eye-level posture (EEP).

To further evaluate robustness under state variation, the dataset was collected under multiple posture conditions, and the present experiments considered four postures, namely P1–P4. As illustrated in Figure 2, the eight gesture classes were Hand Open (HO), Fist (FI), Wrist Flexion (WF), Wrist Extension (WE), Forearm Pronation (FP), Forearm Supination (FS), V-sign (VS), and OK Sign (OK). The four posture conditions were defined as P1: neutral standing posture (NSP), P2: standing posture with 45° shoulder flexion (SF45), P3: standing posture with 90° shoulder flexion (SF90), and P4: elbow-flexed eye-level posture (EEP). This design caused the same gesture to exhibit different muscle recruitment patterns and signal distributions under different upper-limb states, making the dataset closer to practical usage scenarios.

For each LOSO fold, one participant was held out as the target subject. Among the remaining 10 source participants, the next-numbered subject was predefined as the source-validation participant, and the other nine participants were used for source-domain training. This selection followed a fixed cyclic rule; for example, when S1 was the target subject, S2 was used for source validation, whereas when S11 was the target, S1 was used for validation. The rule was fixed before model training and did not depend on target-test performance.

Each gesture was repeated five times under each posture. After retaining the central 5 s of each repetition, each recording contained 2500 samples. With a window length of 150 samples and a step size of 20 samples, each repetition generated 118 windows. For the target subject, rep0 was used exclusively for calibration, whereas rep1–rep4 were reserved for final testing.

Under FT-1_full, all 118 windows from rep0 were used for each of the eight gestures under each of the four postures, resulting in 3776 target calibration windows. Under FT-2_k10, 10 distinct windows were randomly sampled without replacement from the 118 candidate windows of rep0 for each gesture-posture combination using a NumPy random-number generator initialized with a fixed seed of 42. After sampling all eight gestures and four postures, the resulting windows were combined and shuffled using the same random-number generator. Under FT-2_k10, this 320-window set constituted the target calibration pool; 10% was reserved for internal validation during Stage-II fine-tuning, as detailed in Section 4.3. In both settings, the four held-out test repetitions contained 15,104 windows in total.

Importantly, calibration and test partitions were determined at the repetition level before sliding-window segmentation. Windows were then generated independently within each partition, so adjacent or overlapping windows from the same repetition were never divided between target calibration and test sets. Rep0 was fixed across subjects to simulate a standardized first-use calibration scenario, while rep1–rep4 represented subsequent held-out test repetitions.

### 4.2. Data Preprocessing and Windowing

During preprocessing, all filtering operations described in this subsection were implemented digitally after data acquisition. Specifically, because the sampling rate was 500 Hz, a fourth-order Butterworth band-pass filter with an effective passband of 20–237.5 Hz was applied to suppress low-frequency motion artifacts and high-frequency noise. In addition, a 50-Hz notch filter with a quality factor of Q = 30 was used to attenuate power-line interference. Both filters were applied using forward–backward zero-phase filtering to avoid phase distortion. These software-based filters should be distinguished from the pre-ADC analog filtering of the acquisition device, the detailed specifications of which were not disclosed by the manufacturer. After preprocessing, a sliding-window strategy was applied to segment continuous signals into fixed-length local time-series samples for training and evaluation. Specifically, all methods used the same window configuration with a window length of 150 samples and a step size of 20 samples, ensuring a fair comparison across different models and adaptation strategies. At the sampling rate of 500 Hz, this setting corresponded to a temporal window of approximately 300 ms and a step interval of approximately 40 ms. Therefore, each input sample represented a local sEMG segment with an input dimension of 8 × 150. This design preserved short-term temporal dynamics in sEMG signals while maintaining a reasonable balance between sample quantity and computational cost.

### 4.3. Implementation Details

A 1D-CNN was used as the predefined backbone of the proposed framework. ResNet1D, TCN, and CNN-LSTM were evaluated only as auxiliary architecture comparisons under the same cross-subject protocol and were not used as a target-test-driven backbone-selection procedure.

Within each LOSO fold, Stage I used nine source-training participants and one held-out source-validation participant; the target participant was not involved in source-side training or validation. For conventional source pretraining, the Stage-I checkpoint was selected according to Macro-F1 on the held-out source-validation participant, whereas the FOMAML checkpoint was selected according to source-validation meta-task Accuracy. Stage I used the FOMAML task configuration described in Section 3.5. Stage II then fine-tuned the model on the fixed target calibration partition. Augmentation was restricted to the training inputs specified in Section 3.4. Target-test windows were not used as training, validation, or matched samples, and target-test labels were not involved in model or checkpoint selection.

For reproducibility, Stage I was run for 80 epochs with 100 tasks per epoch, an inner-loop learning rate of 0.005, an outer-loop learning rate of 0.001, three inner updates, and a meta-batch size of 4. Stage-II optimization used AdamW with a learning rate of 0.001, a weight decay of 0.0001, a batch size of 256, gradient clipping at 5.0, and label smoothing of 0.05. The Stage-II training schedule was specified separately for the two calibration protocols. Under FT-1_full, fine-tuning was performed for a fixed 80 epochs. Under FT-2_k10, fine-tuning was performed for a maximum of 40 epochs; 10% of the target calibration pool was reserved for internal validation, early stopping with a patience of five epochs was applied, and the checkpoint with the highest validation Accuracy was retained. The random seed was fixed at 42. The corresponding Stage-II schedule was applied consistently to all compared methods within each calibration protocol, and the held-out target-test repetitions were not used for validation or checkpoint selection. MATCHED used the conv1 feature layer, 50 matched windows per class, $\lambda_{0}\;=\;0.02$, a five-epoch warm-up, and subsequent linear annealing toward zero.

### 4.4. Compared Methods

For clarity, the compared methods are divided into four categories:

(1) Training from scratch and adapting directly on a few samples from the target subject;
(2) Source-pretrain + FT, which first trains on source-subject data and then fine-tunes on the target subject;
(3) Source-pretrain + FT + MATCHED, which uses the same conventional source-pretrained initialization as Source-pretrain + FT while applying MATCHED only during Stage-II target-subject fine-tuning;
(4) MAML-based two-stage adaptation, which first learns an initialization through source-subject meta-training and then fine-tunes on the target subject.

Within the fourth category, the individual and combined contributions of data augmentation (AUG) and matched feature regularization (MATCHED) were further investigated.

### 4.5. Evaluation Metrics

Accuracy and Macro-F1 are adopted as the main evaluation metrics, and the final results are reported in terms of mean and standard deviation across subjects. Accuracy reflects the overall classification correctness, while Macro-F1 evaluates classification performance from a class-balanced perspective and thus provides a more comprehensive assessment for multi-class gesture recognition under cross-subject conditions.

## 5. Results and Discussion

Based on the above experimental settings, this section first provides an auxiliary comparison of representative backbone networks while retaining the predefined 1D-CNN for the primary framework. The subsequent analyses evaluate the proposed adaptation strategy under single-posture, FT-1_full, and FT-2_k10 conditions, followed by per-pose, ablation, sensitivity, feature-space, computational-cost, and statistical analyses.

Because the LOSO target subject is the independent experimental unit, the primary pairwise inference was conducted using subject-level paired Wilcoxon signed-rank tests. Each method contributed one paired overall Accuracy and Macro-F1 observation per target subject, and individual sliding windows were not treated as statistically independent observations. Paired mean differences are evaluated together with subject-level Wilcoxon signed-rank tests, matched-pairs rank-biserial correlation ($r_{\mathrm{rb}}$), and Cohen’s $d_{z}$. Two-sided 95% confidence intervals are additionally reported for the comparisons for which they were calculated. The predefined multiplicity family comprised the Accuracy and Macro-F1 comparisons between Source-pretrain + FT and MAML + FT + MATCHED under FT-1_full and FT-2_k10, plus the two additional comparisons between Source-pretrain + FT and Source-pretrain + FT + MATCHED; Holm correction was applied across these six tests. As a complementary cohort-specific analysis of posture-related patterns, a subject-blocked Method × Pose model was fitted with Subject included as a fixed blocking factor. This secondary analysis was used only to examine omnibus Method, Pose, and Method × Pose patterns and was not used to establish pairwise superiority over a specific comparator. For feature-space analysis, testing was performed directly on the original 256-dimensional representations. Features were averaged within each subject × gesture × test-repetition combination before a subject-blocked PERMANOVA with 4999 permutations and corrected pairwise tests.

### 5.1. Comparison of Backbone Networks

**Figure 3:**
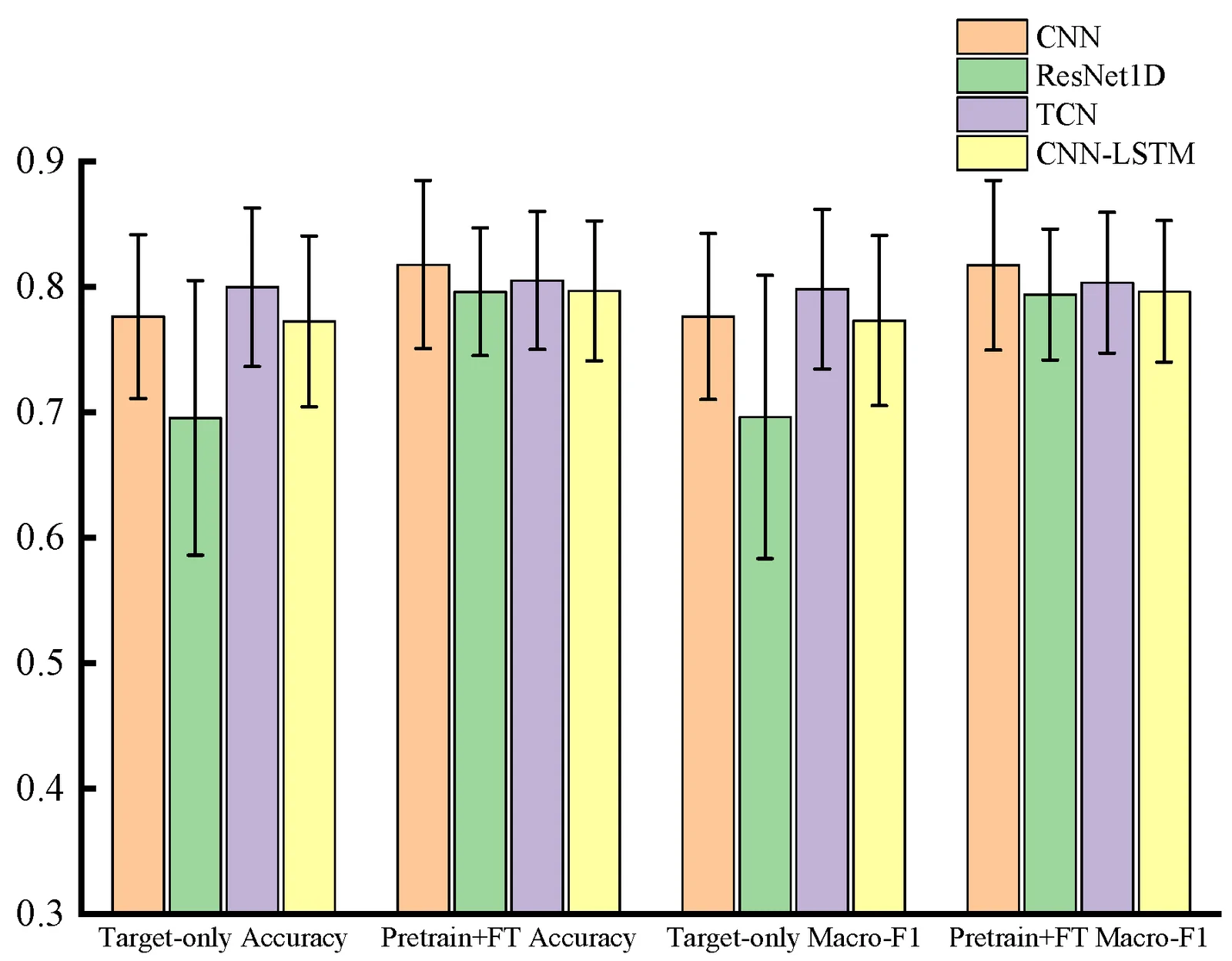
Comparison of different backbone networks under Source-pretrain + FT and target-only adaptation settings.

To provide an auxiliary comparison across representative backbone architectures, CNN, ResNet1D, TCN, and CNN-LSTM were evaluated under Source-pretrain + FT and target-only adaptation. The former used source-domain pretraining before target calibration, whereas the latter adapted from scratch using only target calibration data. The results are shown in Figure 3.

As shown in Figure 3, the predefined CNN produced the highest average Accuracy and Macro-F1 among the evaluated backbones under Source-pretrain + FT (0.8176 ± 0.0669 and 0.8170 ± 0.0676, respectively). This auxiliary comparison characterizes the relative behavior of the architectures under the present protocol and is not interpreted as an independent nested model-selection experiment.

Under target-only adaptation, performance was lower than under Source-pretrain + FT, illustrating the value of source-domain information. TCN showed a slightly higher average than CNN in this target-only comparison. The predefined CNN was retained consistently for all subsequent primary method comparisons.

### 5.2. Results in the Single-Posture Setting (P1)

**Figure 4:**
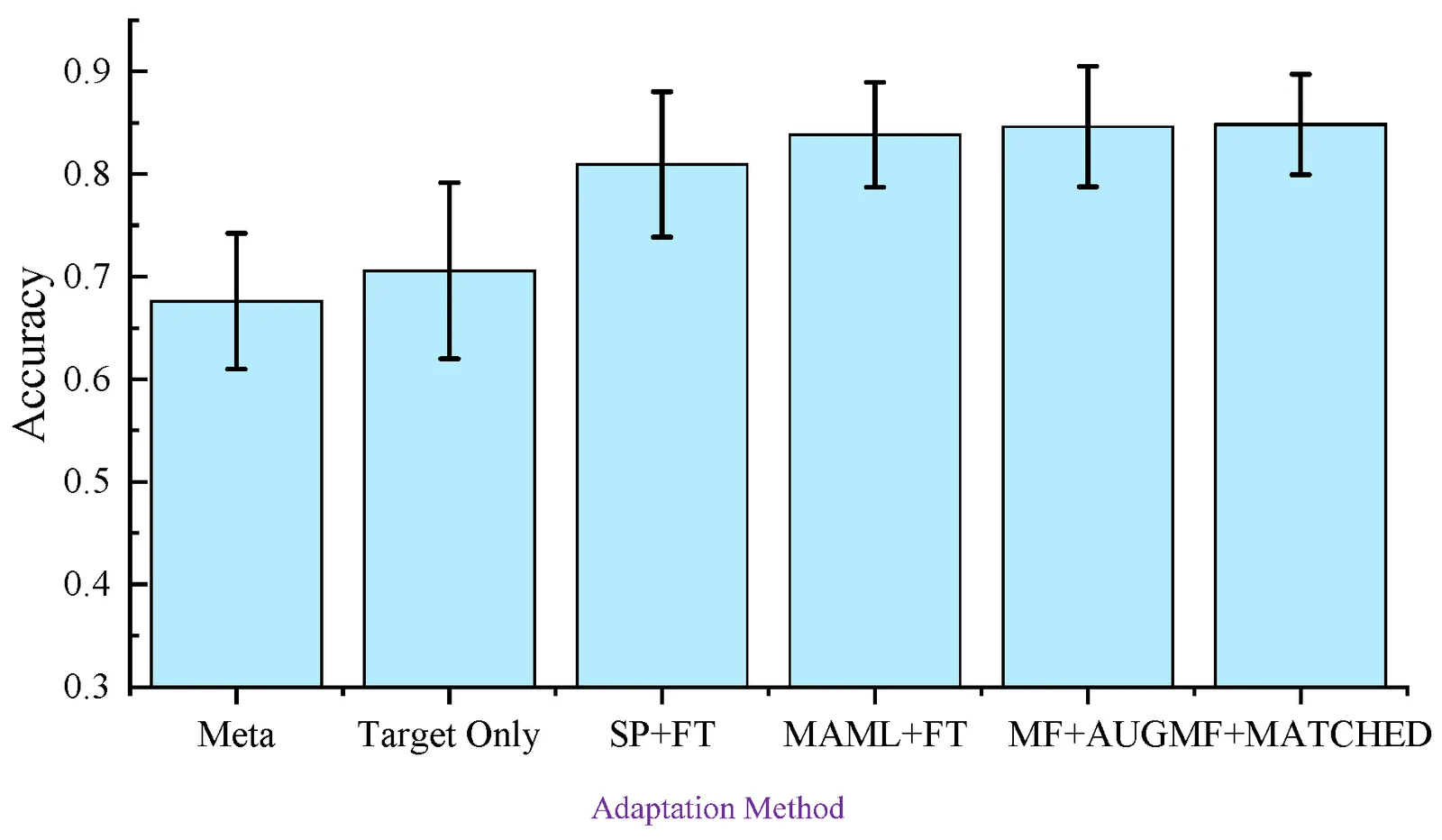
Accuracy comparison of different adaptation methods under the single-posture P1 cross-subject setting. Error bars represent the standard deviation across the 11 LOSO target subjects. Meta denotes Meta Initialization + Adaptation; Target Only denotes adaptation using only target-subject calibration data; SP + FT denotes Source-pretrain + Fine-Tuning; MAML + FT denotes MAML initialization followed by target-subject fine-tuning; MF + AUG denotes MAML + FT + AUG; and MF + MATCHED denotes MAML + FT + MATCHED.

To preliminarily validate the proposed method under a relatively controlled setting, cross-subject experiments were further conducted under the single-posture P1 condition. Compared with the multi-posture setting, posture variation is eliminated here, allowing a more direct assessment of how different adaptation strategies handle cross-subject distribution shifts. The results are shown in Figure 4.

As shown in Figure 4, Target Only yielded the lowest average performance, whereas Source Pretraining + Fine-Tuning produced higher averages. This pattern suggests that source-subject pretraining provided a more informative starting point than learning solely from the small target calibration set under the present P1 protocol.

Meta Initialization + Adaptation alone produced a lower average than Source Pretraining + Fine-Tuning, while the two-stage MAML + Fine-Tuning procedure produced a higher average. These descriptive results support evaluating meta-initialization together with an explicit target fine-tuning stage rather than interpreting meta-initialization alone as sufficient.

Adding augmentation or matched feature regularization to MAML + FT increased the average performance in this single-posture comparison. MATCHED produced the highest average among the displayed configurations, but no pairwise significance claim is made from Figure 4.

### 5.3. Main Results Under FT-1_Full Calibration

**Figure 5:**
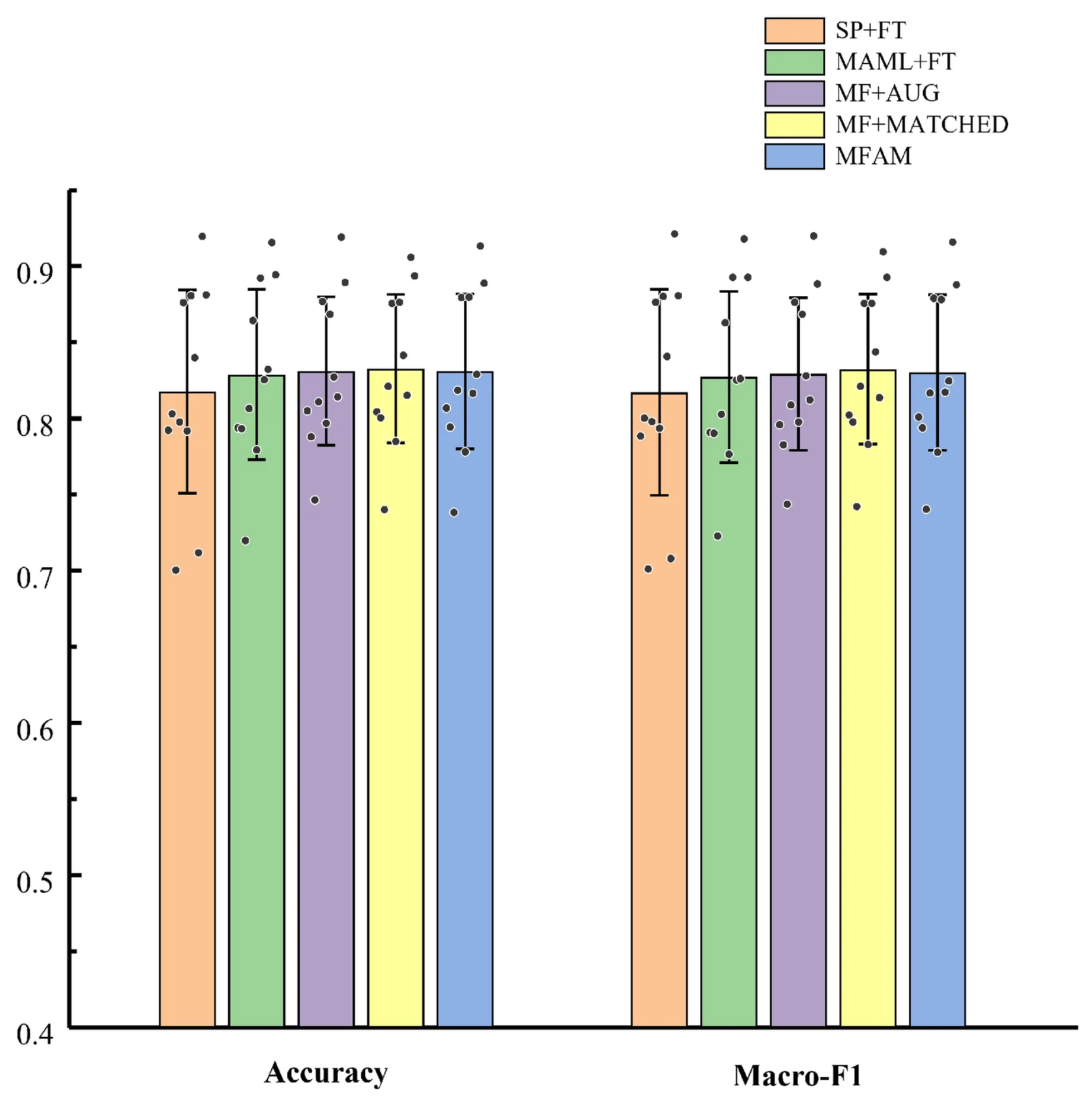
Performance comparison under FT-1_full. Bars represent the mean across 11 LOSO target subjects, error bars indicate ±1 SD, and individual dots denote subject-level results. SP + FT denotes Source-pretrain + Fine-Tuning; MF + AUG denotes MAML + FT + AUG; MF + MATCHED denotes MAML + FT + MATCHED; and MFAM denotes MAML + FT + AUG + MATCHED.

Using the predefined CNN backbone, the adaptation strategies were compared under FT-1_full, in which all 118 windows from target repetition rep0 were used for each gesture and posture. The results are presented in Figure 5.

As shown in Figure 5, all MAML-based fine-tuning configurations produced similar average performance under FT-1_full. The individual-subject points also show that the direction and magnitude of method differences varied across the 11 target subjects.

MAML + FT + MATCHED achieved average Accuracy and Macro-F1 values of 0.8326 and 0.8323, compared with 0.8176 and 0.8170 for Source-pretrain + FT. AUG and MATCHED each produced positive average changes, while their direct combination did not exceed MATCHED alone. Because the average differences were small and heterogeneous across subjects, these results are interpreted descriptively rather than as evidence of an additive or universally superior effect.

For the primary FT-1_full comparison, MAML + FT + MATCHED exceeded Source-pretrain + FT by 1.50 percentage points in Accuracy and 1.53 percentage points in Macro-F1. The paired Wilcoxon tests did not reach statistical significance (Accuracy: *p* = 0.3203, $r_{\mathrm{rb}}$ = 0.3636, $d_{z}$ = 0.3961; Macro-F1: *p* = 0.5771, $r_{\mathrm{rb}}$ = 0.2121, $d_{z}$ = 0.3533). These results indicate positive effect-size trends, while statistically significant pairwise superiority was not established.

To isolate MATCHED from the MAML initialization, Source-pretrain + FT + MATCHED was additionally evaluated under FT-1_full. Adding MATCHED to the conventional source-pretrained initialization increased mean Accuracy from 81.76% to 82.92% and mean Macro-F1 from 81.70% to 82.91%, corresponding to improvements of 1.16 and 1.21 percentage points. The paired 95% confidence intervals were −1.27 to 3.59 and −1.16 to 3.58 percentage points. The Wilcoxon comparisons were not significant (Accuracy: *p* = 0.7002, $r_{\mathrm{rb}}$ = 0.1515, $d_{z}$ = 0.3218; Macro-F1: *p* = 0.6377, $r_{\mathrm{rb}}$ = 0.1818, $d_{z}$ = 0.3430), with Holm-adjusted *p* = 1.0000 for both metrics. This additional comparison indicates a positive but statistically non-significant average contribution of MATCHED under conventional source pretraining.

### 5.4. Results Under FT-2_k10 Calibration

**Figure 6:**
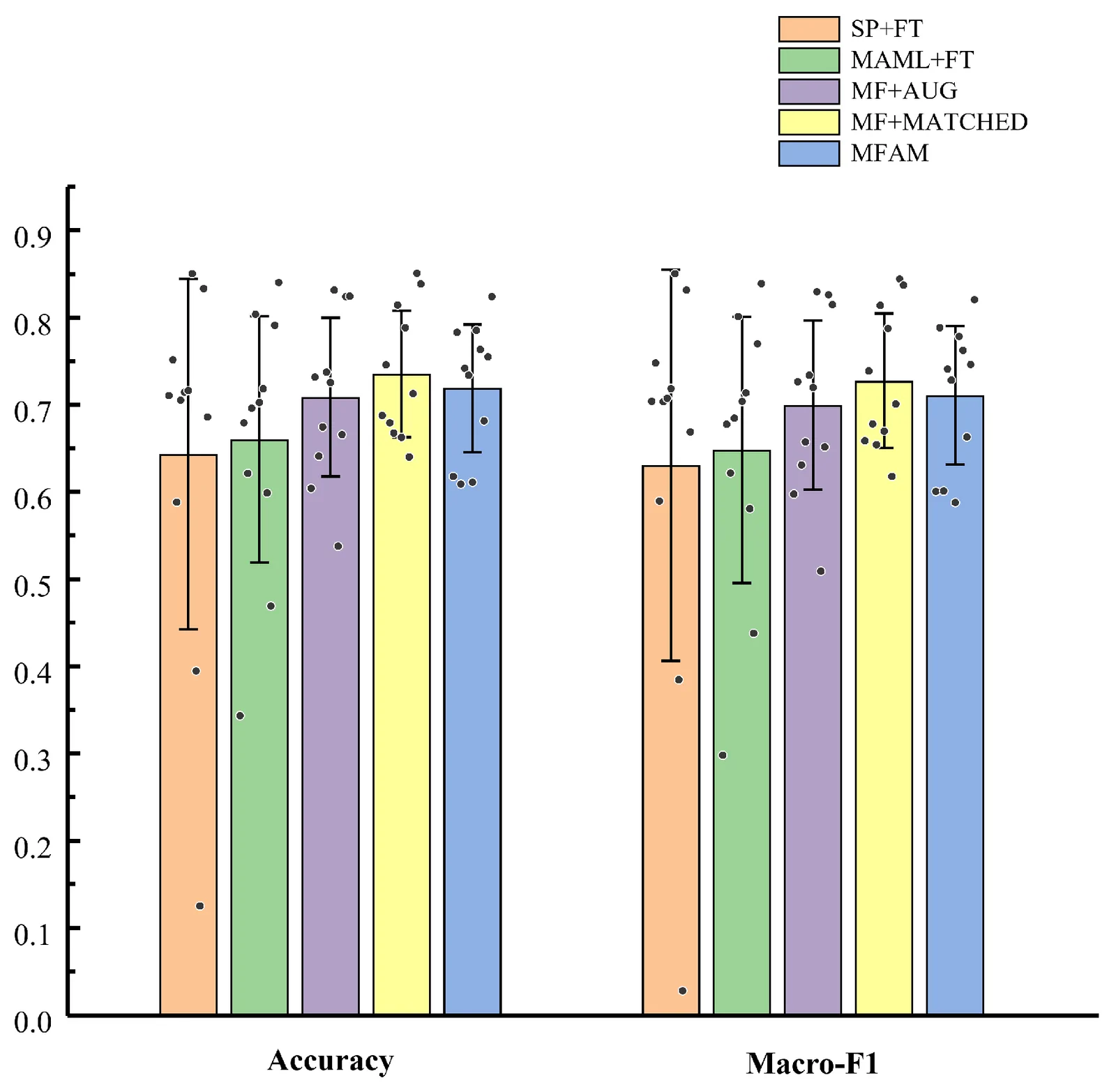
Performance comparison under FT-2_k10. Bars represent the mean across 11 LOSO target subjects, error bars indicate ±1 SD, and individual dots denote subject-level results. SP + FT denotes Source-pretrain + Fine-Tuning; MF + AUG denotes MAML + FT + AUG; MF + MATCHED denotes MAML + FT + MATCHED; and MFAM denotes MAML + FT + AUG + MATCHED.

FT-2_k10 used 10 calibration windows for each gesture under each posture from rep0, corresponding to 320 target calibration windows in total. The held-out test partition was identical to that used under FT-1_full. The results are shown in Figure 6.

Compared with FT-1_full, FT-2_k10 produced greater across-subject dispersion, especially for Source-pretrain + FT and MAML + FT. The individual-subject points show that the low-calibration response was heterogeneous and that no method improved every target subject.

MAML + FT + MATCHED achieved the highest average among the displayed FT-2_k10 configurations, reaching 0.7352 in Accuracy and 0.7273 in Macro-F1. These averages were higher than those of Source-pretrain + FT, MAML + FT, and MAML + FT + AUG, but the uncertainty and subject-level heterogeneity require conservative interpretation.

MAML + FT + MATCHED also showed lower across-subject variability than Source-pretrain + FT. The direct AUG + MATCHED combination did not exceed MATCHED alone on average. This non-additive pattern may reflect partial functional overlap or optimization interference, but the present experiment did not directly test gradient conflict or feature-space damage.

Relative to Source-pretrain + FT, MAML + FT + MATCHED improved mean Accuracy and Macro-F1 by 9.20 and 9.70 percentage points under FT-2_k10. The paired 95% confidence intervals were −3.60 to 22.01 and −4.75 to 24.14 percentage points, respectively. The subject-level Wilcoxon comparisons did not reach significance (Accuracy: *p* = 0.2402, $r_{\mathrm{rb}}$ = 0.4242, $d_{z}$ = 0.4829; Macro-F1: *p* = 0.2402, $r_{\mathrm{rb}}$ = 0.4242, $d_{z}$ = 0.4510), and the Holm-adjusted *p*-values were 1.0000 for both metrics. The improvement is therefore interpreted as a favorable average trend with positive effect sizes rather than a statistically established pairwise gain.

### 5.5. Per-Pose Analysis Under the Multi-Posture Setting

To characterize posture-related performance under both calibration protocols, Table 1 reports the P1–P4 and overall results for Source-pretrain + FT, MAML + FT, MAML + FT + AUG, MAML + FT + MATCHED, and MAML + FT + AUG + MATCHED. Panel A presents FT-1_full and Panel B presents FT-2_k10.

Across both panels of Table 1, average performance varied by posture and calibration level. P4 generally produced lower averages than P1 and P2. Under FT-2_k10, the larger standard deviations for several methods further indicate substantial subject-to-subject variability in low-calibration adaptation.

The per-pose values show descriptive method differences rather than uniform dominance. Under FT-2_k10, MATCHED produced higher average values than AUG on all four postures after correction of the interchanged workbook labels, while the magnitude of the difference varied by posture. These observations are not interpreted as posture-specific pairwise significance because the Method × Pose interaction was not significant.

As a complementary cohort-specific analysis, a subject-blocked Method × Pose model was fitted with Subject included as a fixed blocking factor. Under FT-1_full, the omnibus Method effect was not significant for Accuracy (*p* = 0.5836) or Macro-F1 (*p* = 0.6252). Under FT-2_k10, the omnibus Method effect was significant for Accuracy (*p* = 7.37 × 10^−5^, partial *η*^2^ = 0.1123) and Macro-F1 (*p* = 1.29 × 10^−4^, partial *η*^2^ = 0.1070). The Pose effect was significant under both settings, while the Method × Pose interaction was not significant. This secondary omnibus analysis does not establish that the proposed method was significantly superior to a specific comparator; pairwise claims are based on the primary subject-level Wilcoxon tests.

### 5.6. Effects of AUG and MATCHED Under Expanded Meta-Task Sampling

**Figure 7:**
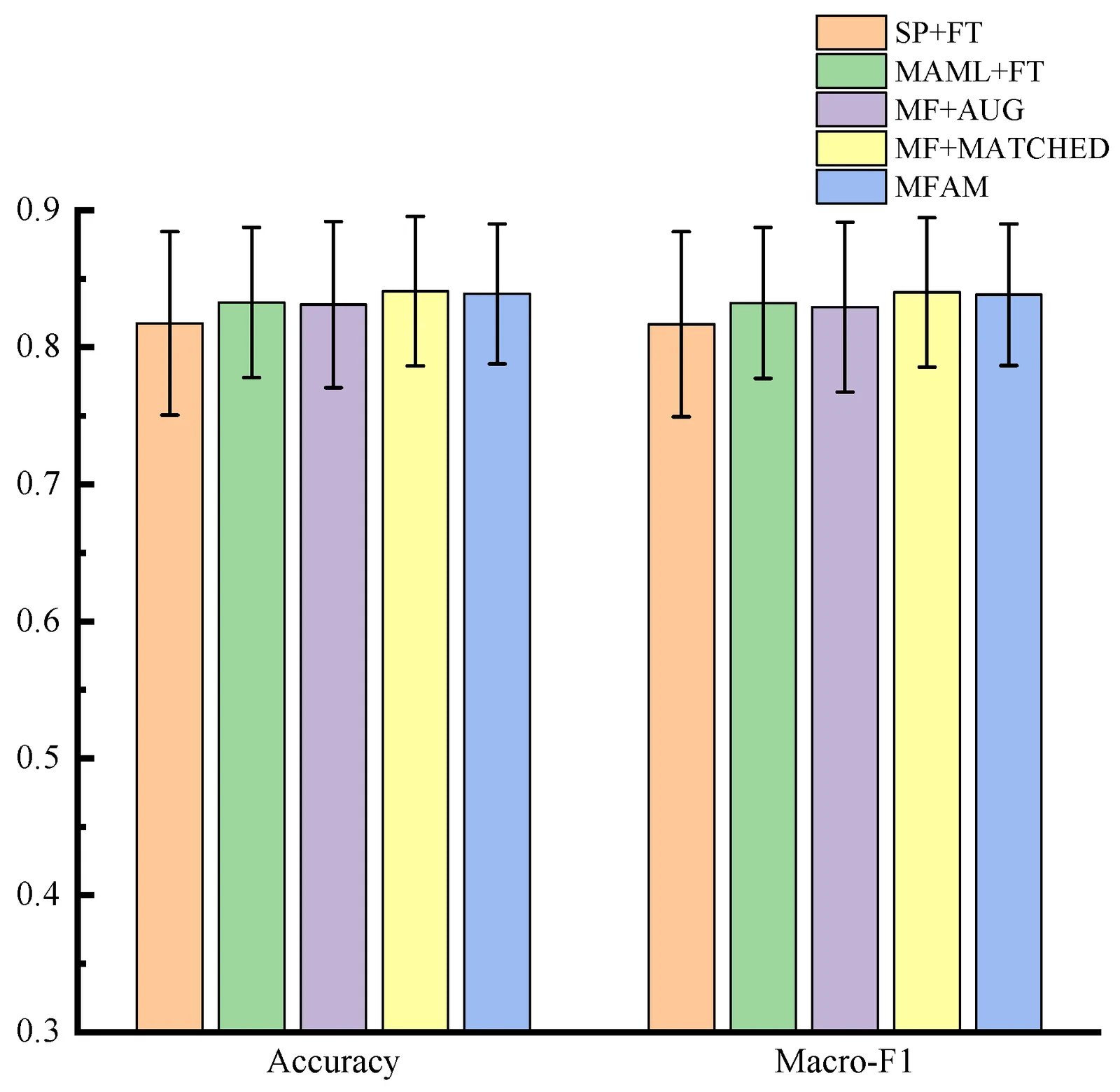
Descriptive contribution analysis of AUG and MATCHED under the expanded meta-task sampling setting. Error bars indicate ±1 SD across the 11 LOSO target subjects.

To examine whether the observed effects of AUG and MATCHED were sensitive to Stage-I task-sampling size, an auxiliary ablation used an expanded meta-task configuration. Compared with the main setting of 10 support and 40 query windows per gesture per posture, the expanded setting used 40 support and 120 query windows per gesture per posture. The target-subject calibration and test protocol remained unchanged. This analysis is therefore a Stage-I sampling-size sensitivity analysis rather than an additional target-data setting. The results are shown in Figure 7.

As shown in Figure 7, both AUG and MATCHED achieved higher average scores than the basic MAML + FT configuration under the current experimental setting, while MATCHED obtained the highest average Accuracy and Macro-F1. However, the direct AUG + MATCHED combination did not yield an additional improvement over MATCHED alone. This observation suggests that the two mechanisms should not be regarded as strictly complementary.

AUG mainly acts at the input level by perturbing signal amplitude, adding low-level noise, and shifting temporal alignment. It therefore encourages invariance to local acquisition variability. In contrast, MATCHED acts at the feature level by reducing the discrepancy between target-domain representations and class-consistent source-domain references. When both terms are active, AUG may enlarge the dispersion of target features, whereas MATCHED attempts to contract those features toward relatively stable reference centroids. These two gradient directions may partially compete, particularly when only a small number of target-domain samples are available.

Importantly, the difference between MATCHED and AUG + MATCHED was limited, and the present ablation does not provide direct statistical evidence that AUG destroys the learned feature space. We therefore interpret the result conservatively as a non-additive interaction or partial functional overlap between input-level perturbation and feature-level regularization.

Overall, MATCHED produced the highest average recognition performance in this expanded-sampling ablation. The AUG + MATCHED configuration did not provide a consistent additional average gain. This result is treated descriptively and does not by itself establish pairwise statistical superiority or a destructive interaction.

### 5.7. Sensitivity Analysis of Matched Regularization Configuration

Table 2 summarizes the sensitivity of matched feature regularization to the alignment layer and the number of matched windows per class under the FT-2_k10 setting. The baseline in Table 2 is the same MAML + FT result under FT-2_k10 as reported in Table 1.

Among the examined configurations, conv1 + wpc50 achieved the highest average Accuracy and Macro-F1, reaching 0.7352 and 0.7273, respectively. Compared with the MAML + FT baseline (0.6604 Accuracy and 0.6480 Macro-F1), these values correspond to descriptive improvements of 7.48 and 7.93 percentage points, respectively. These differences are interpreted descriptively because Table 2 is intended primarily as a configuration-sensitivity analysis.

It is also worth noting that the average effect of increasing the matched-sample scale differed across the examined feature layers. For conv1, increasing the matched scale from wpc25 to wpc50 improved both average Accuracy and Macro-F1. For conv2, the same increase reduced both averages. Under FT-2_k10, these results suggest that the response to matched-sample scale may depend on the alignment layer. One possible explanation is that lower-level conv1 alignment preserves more general temporal and channel-wise sEMG patterns, whereas a stronger constraint at conv2 may restrict feature adaptation; this mechanism was not directly tested in the present study.

To further examine whether the configuration differences were statistically meaningful, additional statistical analyses were conducted based on the subject-level results. The Friedman test across the five configurations showed a significant overall difference in Macro-F1 (*p* = 0.0410), while the difference in Accuracy was not significant (*p* = 0.2438). This indicates that the matched regularization configuration had a more evident influence on class-balanced recognition performance under the FT-2_k10 condition. Pairwise comparisons between conv1 + wpc50 and the other configurations did not remain statistically significant after Holm correction, and therefore conv1 + wpc50 should not be interpreted as being significantly superior to all other configurations.

After excluding the baseline, the Layer × Scale analysis showed interactions for Accuracy (*p* = 0.0296) and Macro-F1 (*p* = 0.0217), indicating that the effect of matched-sample scale depended on the alignment layer. Among the examined configurations, conv1 + wpc50 produced the highest average Accuracy and Macro-F1. Because the analysis was exploratory rather than an independent nested selection procedure, this result is interpreted descriptively and should not be regarded as an unbiased demonstration of an optimal configuration.

### 5.8. Quantitative Feature-Space Analysis

To quantitatively evaluate gesture-class separability in the learned representation, statistical analysis was performed directly in the original 256-dimensional feature space rather than on a low-dimensional embedding.

Features were averaged within each subject × gesture × test-repetition combination, yielding 352 observations from 11 subjects, eight gestures, and four held-out test repetitions. A subject-blocked PERMANOVA with 4999 permutations showed an overall gesture-class effect (pseudo-*F* = 255.26, within-subject $R^{2}$ = 0.9867, *p* = 0.0002). All 28 pairwise gesture comparisons remained significant after Bonferroni and Holm correction ($p_{adj}\;=\;0.0056$), with pairwise $R^{2}$ values ranging from 0.9331 to 0.9906. The PERMANOVA results indicate strong statistical gesture-class separability in the final 256-dimensional representation. Statistical separability at the repetition-aggregated level does not imply complete geometric non-overlap among individual windows. No inferential comparison was performed across the pre-training, meta-training, and fine-tuning stages; therefore, the analysis does not establish a progressive increase in separability across training stages.

### 5.9. Computational Cost Analysis

Table 3 summarizes computational cost. Compared with Source-pretrain + FT, MAML-based meta-training increased Stage-I time from 73.21 s to 446.69 s (approximately 6.1-fold). During Stage-II target adaptation, MAML + FT required 5.54 s and 0.72 s under FT-1_full and FT-2_k10, respectively, whereas MAML + FT + MATCHED required 13.70 s and 1.86 s. MAML + FT + AUG and MAML + FT + AUG + MATCHED required 22.63/2.36 s and 30.54/3.44 s, respectively.

Importantly, all compared methods retained the same 1D-CNN backbone with approximately 370 K parameters and 32.88 M FLOPs per input window. Therefore, although meta-training and matched feature regularization introduced additional training and adaptation costs, they did not increase the parameter count or theoretical inference complexity of the deployed model. In particular, MATCHED alone provided a more favorable performance-cost trade-off than the combined AUG + MATCHED strategy.

### 5.10. Discussion

The present results indicate favorable average performance for the two-stage framework under low-calibration cross-subject adaptation. Meta-learning provides an initialization explicitly optimized for rapid adaptation, while MATCHED adds a class-consistent feature constraint during target fine-tuning. The larger average difference under FT-2_k10 than under FT-1_full suggests that these mechanisms may be more useful when calibration data are scarce; however, the subject-level pairwise tests did not establish statistical superiority over Source-pretrain + FT.

The higher average contribution of MATCHED than AUG in the reported comparisons is consistent with a representation-stabilization interpretation. This interpretation is also compatible with the expanded meta-task sampling ablation, in which MATCHED again produced the highest average. Nevertheless, these analyses do not independently establish the mechanism, and the sensitivity results should be treated as exploratory.

The quantitative feature-space analysis showed strong statistical gesture-class separability in the final 256-dimensional representation. The subject-blocked PERMANOVA revealed a significant overall gesture-class effect, and all 28 pairwise gesture comparisons remained significant after multiple-comparison correction. Nevertheless, statistical separation of the repetition-aggregated representations does not imply complete geometric non-overlap among every individual sEMG window. No inferential comparison was performed across the pre-training, meta-training, and fine-tuning stages; therefore, this analysis does not establish progressive stage-wise enhancement of class separability.

The two statistical analyses address different questions. The subject-level Wilcoxon tests provide the primary inference for predefined pairwise method comparisons, whereas the subject-blocked Method × Pose analysis provides complementary information about omnibus method- and posture-related patterns within the present cohort. Accordingly, the significant omnibus Method effect under FT-2_k10 should not be interpreted as evidence that the proposed method was significantly superior to a specific comparator.

The effect-size analysis further clarifies the scope of the observed improvements. Although the subject-level pairwise Wilcoxon tests did not reach statistical significance, the corresponding effect-size estimates were positive. Under FT-2_k10, MAML + FT + MATCHED showed a larger average improvement over Source-pretrain + FT than under FT-1_full. Therefore, the present results should be interpreted as favorable average-performance and effect-size trends rather than universal pairwise statistical superiority.

The posture-wise results provide additional descriptive context. P4 was generally the most difficult posture, whereas P1 and P2 produced higher averages, indicating that posture-related difficulty was not uniform across upper-limb states. MATCHED showed numerically larger average gains than AUG under the reported comparisons. However, the Method × Pose interaction was not significant, so these data do not establish a posture-specific benefit or prove that the relative method effects are identical across postures.

The effects of input-level augmentation and matched feature regularization were not strictly additive in the present experiments. AUG introduced controlled variations in signal amplitude, low-level noise, and temporal alignment, thereby encouraging the model to become less sensitive to local acquisition variability. MATCHED, in contrast, constrained target-domain representations toward class-consistent source-domain references during fine-tuning. When both mechanisms were applied simultaneously, augmentation tended to enlarge the local variation in the target inputs, whereas matched regularization tended to reduce the feature discrepancy between the target and source reference samples. These different optimization objectives provide a plausible explanation for why AUG + MATCHED did not consistently outperform MATCHED alone.

Nevertheless, the observed performance difference between MATCHED and AUG + MATCHED was limited, and the present experiments did not directly evaluate gradient conflict or demonstrate that augmentation damaged the learned feature space. Therefore, the non-additive result should be interpreted conservatively as possible functional overlap or mild optimization interference rather than as direct evidence of destructive noise. Future work may investigate separately coordinated classification and regularization branches, adaptive augmentation strength, or gradient-level analyses to better understand and reduce this interaction.

From a computational perspective, the proposed framework increased offline meta-training and target-subject adaptation costs. However, all methods retained the same 1D-CNN backbone with approximately 370 K parameters and 32.88 M FLOPs per input window, and the matched regularization branch was not retained during inference. Therefore, the proposed strategy should be regarded as preserving lightweight inference rather than as having negligible training overhead.

From an application perspective, the framework retains the same inference network as the underlying 1D-CNN, although it increases offline training and adaptation cost. The present study has several limitations. Evaluation was restricted to a self-collected 11-subject dataset and a fixed rep0 calibration protocol. Rotating the calibration repetition would provide a more complete estimate of repetition-to-repetition variability. Validation on public datasets, independent cohorts, and cross-session, cross-day, and cross-device recordings is required before broader generalizability can be established. In addition, the examined matched-regularization configuration was evaluated through an exploratory sensitivity analysis; future work should use nested source-domain validation to select matching hyperparameters independently of the final target-subject evaluation. Under FT-2_k10, the present evaluation used a single predefined support-set realization generated with a fixed random seed of 42. Although this provides a consistent low-calibration setting for method comparison, it does not quantify sensitivity to alternative support-set draws. Because adjacent candidate windows originate from the same repetition and may be highly overlapping, repeated evaluation across independently sampled support sets would provide a more complete assessment of sampling variability in future work.

One limitation of the present study is that the acquisition device uses a proprietary and non-open-source analog front end. Although the available electrode, sampling, amplification, and noise specifications were verified with the manufacturer, the exact input impedance, CMRR, and pre-ADC analog filtering parameters were not disclosed. These unavailable hardware details may limit complete replication of the acquisition circuitry, although the electrode configuration, physical placement, artifact-control procedures, sampling settings, and digital preprocessing pipeline have now been reported in detail.

## 6. Conclusions

This study investigated cross-subject sEMG gesture recognition under posture-varying and low-calibration conditions. A two-stage adaptation framework was developed by combining MAML-based source-subject meta-training, target-subject fine-tuning, training-time data augmentation, and matched feature regularization. MAML was used to learn an initialization suitable for rapid adaptation, while matched source-domain samples provided an auxiliary class-consistent feature constraint during target-subject fine-tuning.

Experiments on a self-collected multi-posture sEMG dataset under LOSO showed favorable average Accuracy and Macro-F1 for the MAML-based methods relative to conventional Source-pretrain + FT. Under FT-1_full, MAML + FT + MATCHED improved the average Accuracy and Macro-F1 by 1.50 and 1.53 percentage points, respectively, although the corresponding subject-level Wilcoxon comparisons did not reach statistical significance. Under FT-2_k10, the average improvements were 9.20 and 9.70 percentage points, with paired 95% confidence intervals of −3.60 to 22.01 and −4.75 to 24.14 percentage points. The corresponding Wilcoxon tests did not reach statistical significance (*p* = 0.2402 for both FT-2_k10 metrics; Holm-adjusted *p* = 1.0000). Pairwise statistical superiority was therefore not established, although the effect-size estimates and average trends were positive.

The ablation results indicated that matched feature regularization achieved the highest average performance under the examined configuration. The results also showed that AUG and MATCHED were not strictly additive. AUG introduced controlled input-level perturbations, whereas MATCHED constrained target features toward class-consistent source references. Their direct combination did not consistently outperform MATCHED alone, suggesting possible functional overlap or mild optimization interference. The current experiments did not directly demonstrate destructive noise or feature-space degradation, and this interpretation should therefore remain cautious.

Overall, the results support further investigation of meta-learned initialization combined with training-time feature regularization for low-calibration cross-subject sEMG adaptation. The matched samples are used only during fine-tuning, so the deployed inference network remains the same approximately 370 K-parameter, 32.88 M-FLOP 1D-CNN. The conclusions are limited by the 11-subject self-collected dataset, the fixed-repetition LOSO protocol, the absence of independent public-dataset validation, the exploratory configuration analysis, and unavailable proprietary analog-front-end specifications.

Future work will evaluate the framework on larger public and independently collected datasets, rotate the calibration repetition, and test cross-session, cross-day, and cross-device conditions. Nested source-domain validation will be used for hyperparameter selection, together with adaptive matched-sample selection and separately coordinated augmentation and regularization branches.

## Figures and Tables

**Table 1:** Per-pose recognition results under FT-1_full (Panel A) and FT-2_k10 (Panel B). Values are mean ± SD across the 11 LOSO target subjects.

**Panel A. FT-1_full**						
**Method**	**Metric**	**P1**	**P2**	**P3**	**P4**	**Overall**
Source-pretrain + FT	Accuracy	0.8387 ± 0.0647	0.8357 ± 0.0725	0.8185 ± 0.0763	0.7774 ± 0.1059	0.8176 ± 0.0669
	Macro-F1	0.8365 ± 0.0674	0.8323 ± 0.0750	0.8139 ± 0.0801	0.7740 ± 0.1074	0.8170 ± 0.0676
MAML + FT	Accuracy	0.8564 ± 0.0470	0.8501 ± 0.0648	0.8234 ± 0.0700	0.7853 ± 0.0924	0.8288 ± 0.0560
	Macro-F1	0.8522 ± 0.0524	0.8472 ± 0.0672	0.8196 ± 0.0722	0.7786 ± 0.0956	0.8273 ± 0.0563
MAML + FT + AUG	Accuracy	0.8560 ± 0.0430	0.8583 ± 0.0549	0.8286 ± 0.0657	0.7815 ± 0.0895	0.8311 ± 0.0488
	Macro-F1	0.8525 ± 0.0464	0.8550 ± 0.0570	0.8234 ± 0.0689	0.7776 ± 0.0923	0.8292 ± 0.0502
MAML + FT + MATCHED	Accuracy	0.8514 ± 0.0449	0.8555 ± 0.0579	0.8284 ± 0.0572	0.7950 ± 0.0847	0.8326 ± 0.0487
	Macro-F1	0.8477 ± 0.0493	0.8537 ± 0.0611	0.8261 ± 0.0582	0.7919 ± 0.0864	0.8323 ± 0.0492
MAML + FT + AUG + MATCHED	Accuracy	0.8541 ± 0.0463	0.8568 ± 0.0597	0.8275 ± 0.0792	0.7864 ± 0.0977	0.8312 ± 0.0555
	Macro-F1	0.8514 ± 0.0494	0.8542 ± 0.0630	0.8232 ± 0.0808	0.7846 ± 0.0978	0.8305 ± 0.0558
**Panel B. FT-2_k10**						
**Method**	**Metric**	**P1**	**P2**	**P3**	**P4**	**Overall**
Source-pretrain + FT	Accuracy	0.6792 ± 0.2064	0.6523 ± 0.2198	0.6387 ± 0.1973	0.6026 ± 0.2056	0.6432 ± 0.2010
	Macro-F1	0.6618 ± 0.2305	0.6355 ± 0.2417	0.6191 ± 0.2210	0.5861 ± 0.2272	0.6303 ± 0.2244
MAML + FT	Accuracy	0.7126 ± 0.1201	0.6861 ± 0.1336	0.6435 ± 0.1477	0.5992 ± 0.1894	0.6604 ± 0.1412
	Macro-F1	0.6894 ± 0.1378	0.6717 ± 0.1462	0.6251 ± 0.1626	0.5769 ± 0.2015	0.6480 ± 0.1526
MAML + FT + AUG	Accuracy	0.7384 ± 0.0841	0.7331 ± 0.0996	0.7171 ± 0.0906	0.6466 ± 0.1325	0.7088 ± 0.0911
	Macro-F1	0.7239 ± 0.0906	0.7223 ± 0.1078	0.7051 ± 0.0964	0.6245 ± 0.1488	0.6997 ± 0.0972
MAML + FT + MATCHED	Accuracy	0.7772 ± 0.0462	0.7600 ± 0.0870	0.7295 ± 0.0931	0.6743 ± 0.1200	0.7352 ± 0.0726
	Macro-F1	0.7661 ± 0.0530	0.7519 ± 0.0917	0.7170 ± 0.0953	0.6550 ± 0.1317	0.7273 ± 0.0772
MAML + FT + AUG + MATCHED	Accuracy	0.7484 ± 0.0823	0.7510 ± 0.0866	0.7172 ± 0.0751	0.6581 ± 0.1041	0.7187 ± 0.0731
	Macro-F1	0.7276 ± 0.0972	0.7374 ± 0.0960	0.7043 ± 0.0792	0.6430 ± 0.1110	0.7108 ± 0.0794

**Table 2:** Exploratory sensitivity analysis of matched feature regularization under FT-2_k10. MAML + FT (Baseline) is the same FT-2_k10 MAML + FT result reported in Table 1; wpc25 and wpc50 denote 25 and 50 matched windows per class; bold indicates the highest average value.

Alignment Layer	Matched Scale	Accuracy	Macro-F1
MAML + FT (Baseline)		0.6604 ± 0.1412	0.6480 ± 0.1526
conv1	wpc25	0.7178 ± 0.0679	0.7092 ± 0.0753
conv1	wpc50	0.7352 ± 0.0726	0.7273 ± 0.0772
conv2	wpc25	0.7299 ± 0.0975	0.7195 ± 0.1081
conv2	wpc50	0.6712 ± 0.1186	0.6551 ± 0.1280

**Table 3:** Computational cost of the compared adaptation strategies under FT-1_full and FT-2_k10.

Method	Stage-I Time (s)	Stage-II FT-1_full/FT-2_k10 (s)	Params	FLOPs/Window
SP + FT	73.21	5.53/0.69	370 K	32.88 M
MAML + FT	446.69	5.54/0.72	370 K	32.88 M
MAML + FT + AUG	446.69	22.63/2.36	370 K	32.88 M
MAML + FT + MATCHED	446.69	13.70/1.86	370 K	32.88 M
MAML + FT + AUG + MATCHED	446.69	30.54/3.44	370 K	32.88 M

## Data Availability

The data presented in this study are available from the corresponding author upon reasonable request. The raw sEMG data are not publicly available due to privacy and ethical restrictions related to human-subject signal recordings.
